# Synthesis and Characterization of Cu_2_FeSnS_4_–Cu_2_MnSnS_4_ Solid Solution Microspheres

**DOI:** 10.3390/ma13194440

**Published:** 2020-10-07

**Authors:** Edyta Waluś, Maciej Manecki, Grzegorz Cios

**Affiliations:** 1 Department of Mineralogy, Petrography, and Geochemistry, AGH University of Science and Technology, al. Mickiewicza 30, 30-059 Kraków, Poland; gpmmanec@cyf-kr.edu.pl; 2 Academic Centre for Materials and Nanotechnology, AGH University of Science and Technology, 30-059 Kraków, Poland; ciosu@agh.edu.pl

**Keywords:** stannite, CFTS, CMTS, chalcogenides, hydrothermal synthesis, solar cell

## Abstract

In this study, we used a hydrothermal method to synthesize microspheres of Cu_2_(Mn_1−x_Fe_x_)SnS_4_ solid solution (X = 1, 0.8, 0.6, 0.4, 0.2, 0). The process was optimized to improve the crystallinity, morphology, and purity of the obtained materials. All samples were characterized by X-ray diffraction (XRD), scanning electron microscopy (SEM), energy-dispersive X-ray spectroscopy (EDS), Raman spectroscopy, and Fourier transform infrared (FTIR) spectroscopy. The following conditions were optimized: A mixture of water and ethylene glycol at the ratio of 1:7 as the reaction medium, polyvinylpyrrolidone (PVP) as the surface ligand, and reaction temperature of 195 °C for 7 days. The product of synthesis precipitated in the form of aggregates of nanocrystals, which form homogeneous, often concentric microspheres with a diameter of 1–1.5 μm. The chemical composition of the product can be well controlled by the chemical composition of the reactants. The compound Cu_2_(Mn_1−x_Fe_x_)SnS_4_ forms a continuous series of solid solutions.

## 1. Introduction

In the current era of thin-film solar cells, it is necessary to produce low-cost, high-performance single-connector solar cells with high conversion efficiency. In contrast to most thin-film solar technologies, chalcopyrite-based thin solar cells using CuIn_x_Ga_1−x_S(Se)_2_ (CIGS) solar panels offer a potentially competitive efficiency to traditional silicon panels [1,2,3]. The highest efficiency of this material achieves 23.35 [4], but they are currently very expensive to produce and the components are rare and not environment friendly. Therefore, quaternary chalcogenide semiconductors of I_2_-II-IV-VI_4_ series (where I = Cu and Ag; II = Zn, Cd, Fe, and Mn; IV = Si, Ge, and Sn; and VI = S and Se) have drawn great interest as an alternative absorber material in thin film solar cells that have reached 10% efficiency, and contains only nontoxic and Earth-abundant elements. They have potential applications not only as photovoltaic materials [5,6,7,8,9,10,11,12,13].

In this group, extensive research has been conducted on kesterites (Cu_2_ZnSnS_4_ (CZTS)) and stannites (Cu_2_FeSnS_4_ (CFTS)) [14,15]. Extensive characterization of the synthetic stannite-type material namely, Cu_2_MnSnS_4_ (CMTS), has also been conducted. Similar to CFTS, CMTS is also considered as possible photovoltaic materials, due to its suitable bandgap and the requisite optical characteristics [16]. Several methods have been developed to fabricate these materials [17,18,19,20,21,22,23,24,25,26,27,28,29,30,31,32,33,34,35,36]. A previous review article has described various methods to achieve this goal [37], which need different synthesis times (for example liquid reflux method takes 6–12 h [23], synthesized via microwave needs irradiatiion for 5 min [18] or sonochemical synthesis takes 3 h [38]). Some of these techniques require additional thermal annealing [28], which takes extra time. There is no consensus on the optimal processing method. Among these methods, the hydrothermal method has been widely used due to a simpler procedure, which is eco-friendly and economical in nature. Hydrothermal reactions have been successfully employed in the synthesis of various chemicals. A number of microporous solids have been synthesized using hydrothermal reactions. Various groups of researchers, such as geologists and mineralogists have also been able to determine the conditions necessary for their formation in nature. The use of hydrothermal synthesis facilitates the production of crystals of substances that are usually unstable near the melting point. In addition, it allows for the synthesis of large crystals with high quality. Hydrothermal synthesis can be effective both, at temperatures and pressures below the critical point for a specific solvent and under supercritical conditions. This method allows to control the chemical composition of the final product. One of the disadvantages of this method is the inability to monitor crystals during the process of their growth. Despite this, it is still popular among the industries producing nanomaterials. Although, several studies have used the conventional or microwave solvothermal/hydrothermal route for the synthesis of CZTS or CFTS, this technique still requires more work and optimization [37]. In a typical procedure, the synthesis of quaternary chalcogenide semiconductors takes 24 h [39].

Most I_2_–II–IV–VI_4_ compounds crystallize with a zinc-blende (ZB) or wurtzite (WS)-type superstructure, showing the tetragonal stannite (ZB-derived) or the orthorhombic wurtzite–stannite (WS-derived) structure [1]. The crystal structure of phases in the solid solution series of Cu_2_FeSnS_4_–Cu_2_MnSnS_4_ has been thoroughly examined in a previous study [40]. The authors determined a continuous solid solution in the series. The structure was described in the tetragonal system (the space group I–42m), where each cation is tetrahedrally coordinated to four sulfur anions in a sphalerite-like arrangement. However, several previous investigations have indicated that the formation of a certain crystal structure can be closely related to the reaction conditions [see 1 and the literature cited, therein]. So far, the methods of synthesis of CFTS–CMTS solid solution series are poorly studied. In a previous study [40], the material was synthesized using quartz ampoules sealed under Ar atmosphere and heated to 850 °C for about 72 h. This method may have little use in future industrial applications.

In this study, we prepared a series of compounds of Cu_2_(Mn_1−x_Fe_x_)SnS_4_ (X = 1, 0.8, 0.6, 0.4, 0.2, 0) in the form of spherical microparticles by using the hydrothermal synthesis method requiring simple reactants and relatively low temperature. We experimentally identified the optimal conditions for the synthesis of these compounds. This method the morphology and chemical composition of the resulting material to be controlled, which precipitates in the form of uniform microspheres composed of nanocrystals. It contributes to the development of production of photovoltaic materials with the required stoichiometry by means of a convenient synthesis technique by controlling the ratio of precursors.

## 2. Materials and Methods

### 2.1. Materials

Analytical grade ethylene glycol (EG), copper (II) chloride dihydrate (CuCl_2_⋅2H_2_O), tin (IV) chloride pentahydrate (SnCl_4_⋅5H_2_O), ferrous chloride tetrahydrate (FeCl_2_⋅4H_2_O), manganese chloride tetrahydrate (MnCl_2_⋅4H_2_O), thiourea (Tu), polyvinylpyrrolidone (PVP, Mw = 55 g/mol), and double distilled water were used in the synthesis. All chemicals were purchased from Sigma Aldrich (Merck, Darmstadt, Germany) and were used as received.

### 2.2. Synthesis of Microspheres

The solutions of 1 mmol copper (II) chloride dihydrate, 0.5 mmol tin (IV) chloride pentahydrate, 0.5 mmol ferrous chloride tetrahydrate, 0.5 mmol manganese chloride tetrahydrate, and 2.5 mmol Tu were mixed in the proportions resulting in a molar ratio of Cu, (Mn + Fe), Sn, and S equal to 2:1:1:4. To obtain solutions with different content of Fe and Mn, the molar ratio of Fe/(Mn + Fe) in the solution was varied (0.0, 0.2, 0.4, 0.6, 0.8, and 1). The solution was mixed with 1.68 g PVP, 24–96 mL EG, and 0–72 mL double distilled water under magnetic stirring. Various concentrations of EG and H_2_O were prepared in different ratios: 1:0, 7:1, 3:1, 5:3, 1:1, 3:5, and 1:3. The mixture (96 mL) was transferred to two Teflon-lined containers and maintained at various temperatures (in the range of 160–195 °C) for various time intervals (from 1 to 14 days). The products were washed several times with water and acetone, centrifuged, and air-dried at 60 °C.

### 2.3. Characterization of Microspheres

The morphology of CFTS microspheres was characterized by using scanning electron microscopy (SEM, FEI Quanta 200 FEG and FEI Sirion 200, FEI, Hillsboro, OR, USA) equipped with secondary electron (SE) and back-scattered electron (BSE) detectors. An energy dispersive spectrometer (EDS, FEI Quanta (FEI, Hillsboro, OR, USA) was employed to monitor variations in the chemical composition of the microspheres. Samples of microspheres were fixed on a substrate with double-sided sticky tape and imaged without coating in a low vacuum. The selected samples were immersed in epoxy and polished to reveal the internal structure of the particles. Powder X-ray diffraction (XRD) patterns were recorded with Rigaku SmartLab diffractometer (Neu-Isenburg, Tokyo, Japan) in the range of 2–75° 2θ with a step size of 0.05° using graphite-monochromatized Cu Kα radiation. The phases were identified using the International Centre for Diffraction Data (ICDD) database and XRAYAN software (v 4.0.5, “KOMA” – Henryk Marciniak, Warszawa, Poland) [41]. For Raman spectroscopy, Thermo Scientific DXR Raman Microscope (Thermo Fisher Scientific, Waltham, MA, USA) was used. Spectra were recorded at room temperature with a green laser (k = 532 nm, laser power of 10 mW, slit aperture of 25 μm, resolution of 1.9 cm^−1^ in the range between 100 and 3579 cm^−1^). A total of 10 exposures of 3 s were taken for each spectrum. Spectra were interpreted with the aid of OMNIC for Dispersive Raman software (v 8.3.103, Thermo Fisher Scientific, Waltham, MA, USA). Infrared spectra were acquired using Bruker Sensor 27 spectrometer (BRUKER, Ettlingen, Germany) in the range of 400–4000 cm^−1^ (64 scans at the resolution of 1 cm^−1^). Prior to analysis, KBr pellets were prepared by homogenizing 200 mg of ground KBr with 4 mg of the sample.

## 3. Results and Discussion

### 3.1. Optimization of the EG:H_2_O Ratio

To obtain the optimum condition for the formation of isomorphic series of Cu_2_(Mn_1−x_Fe_x_)SnS_4_ microspheres, the effect of the reaction conditions (including the ratio of EG to H_2_O, the reaction temperature, and duration of the synthesis) on the morphological and structural properties of as-synthesized CFTS and CMTS microspheres was analyzed. In the next step, the optimum conditions were chosen to synthesize and characterize all the members of the stannite isomorphic series with Mn-Fe substitutions.

**Figure 1:**
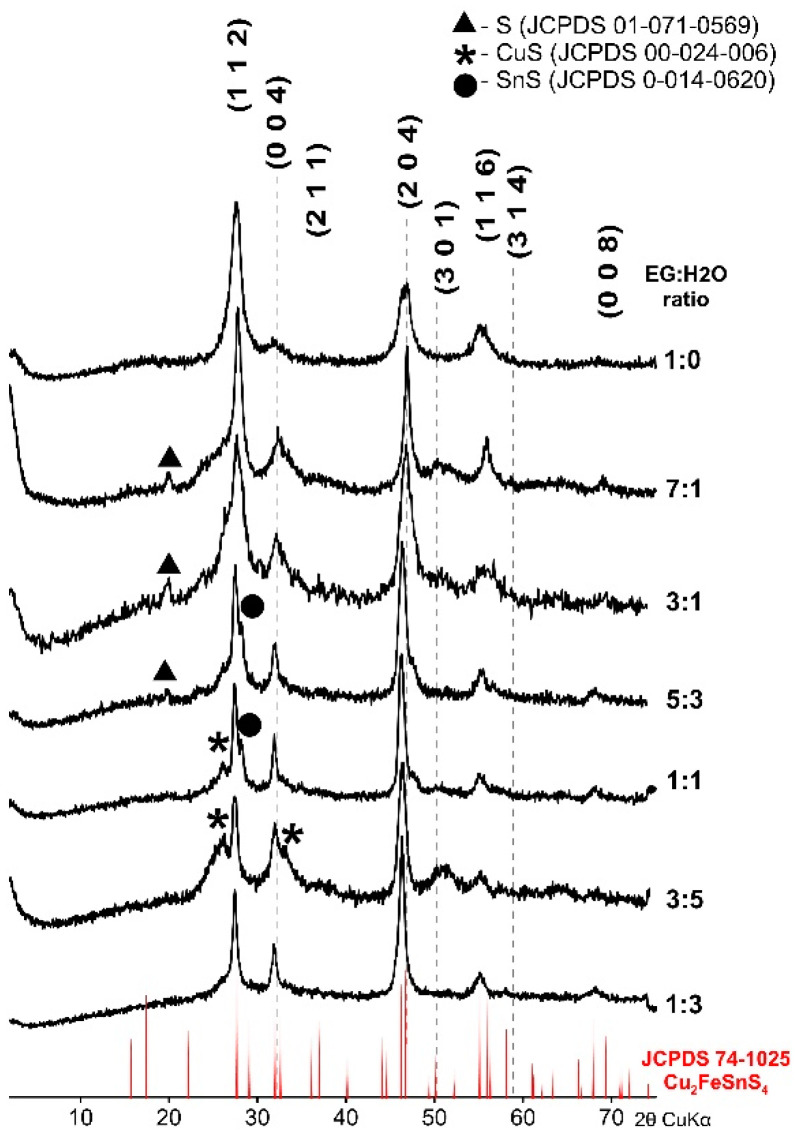
Powder X-ray diffraction pattern of Cu_2_FeSnS_4_ (CFTS) nanoparticles synthesized at various EG:H_2_O ratios with the stannite standard positions marked at the bottom. In some samples impurities were detected as artifacts of an incomplete reaction. The optimal EG:H_2_O ratio is 7:1.

CFTS synthesized for 1 week at 195 °C with different proportions of EG and H_2_O were characterized by XRD and SEM-EDS. Figure 1 shows the diffraction patterns of the products of the synthesis. All XRD patterns are strongly influenced by the formation of small crystallites and amorphization. The position of main diffraction peaks in all samples correspond to the tetragonal structure of CFTS stannite (JCPDS 74-1025). In some samples, small admixtures of elementary sulfur, CuS, or SnS were detected as artifacts of incomplete reaction. The most intensive patterns with the sharpest peaks and the best match of reflections to the standard stannite pattern resulted from the EG:H_2_O ratio equal to 1:0 and 7:1, respectively. The products indicate the desired CFTS with the best crystallinity. Products synthesized from EG:H_2_O ratio of 3:1, 5:3. 1:1, and 3:5 show a good match of reflections with standard patterns but contain more impurities. Furthermore, the products synthesized from EG:H_2_O at a ratio of 1:3 consisted of a single-phase, but the peaks were of low intensity and their position was shifted toward the lower values of 2θ.

**Figure 2:**
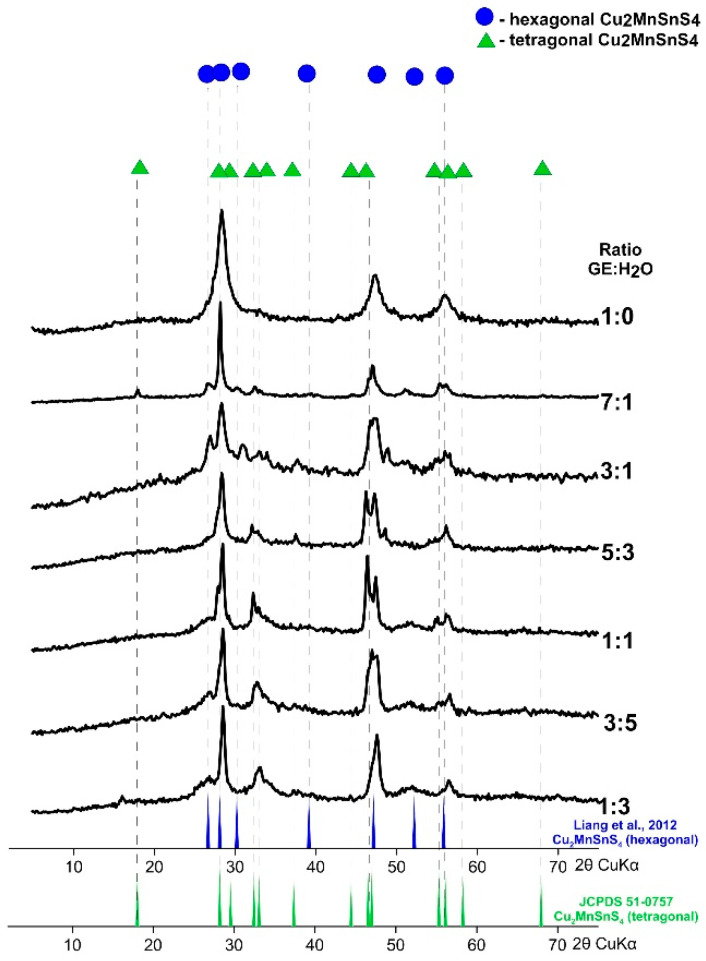
Powder X-ray diffraction pattern of Cu_2_MnSnS_4_ (CMTS) nanoparticles synthesized at various EG:H_2_O ratios. As the water content increases, the peaks characteristic of the hexagonal structure become clearer.

XRD patterns of CMTS obtained at different ratios of EG:H_2_O by synthesis lasting for 1 week at 195 °C are compared in Figure 2. The synthesis in pure glycol (EG:H_2_O = 1:0) leads to the formation of a tetragonal CMTS (JCPDS card no. 51-0757). With the ratio EG:H_2_O = 7:1 a product with mixed tetragonal and hexagonal structure forms. The position of the reflexes at 2θ° = ~18.02, 28.21, 29.56, 32.47, 33.07, 37.37, 44.46, 46.99, 55.31, correspond to the tetragonal structure (JCPDS 51-0757) and the positions at 2θ° = ~26.77, 28.21, 30.32, 39.23, 47.23, 52.26, 55.89 correspond to the hexagonal structure (Liang et al., 2012). The same can be observed for EG:H_2_O = 3:1. However, in this case the peaks are less intense and broad. As the water content increases, the peaks characteristic of hexagonal CMTS become clearer which indicates that the crystalline structure of CMTS is gradually transforming. Note that the optical band gaps for tetragonal CMTS and hexagonal CMTS are almost the same (ca. 1.1 eV as calculated by Liang et al. [42]) suggesting that both structures have the same potential application in solar cells.

**Figure 3:**
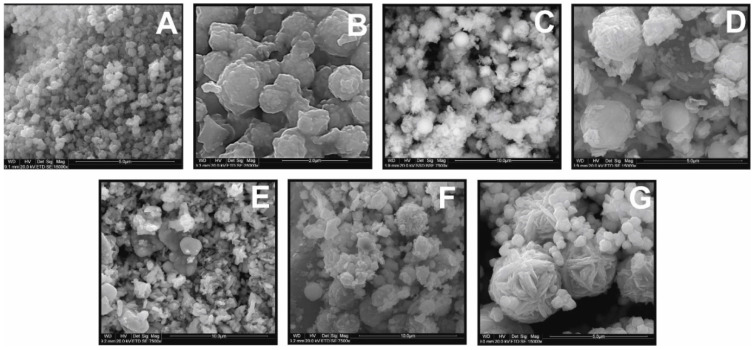
The effect of various EG:H_2_O ratios on the morphology of Cu_2_FeSnS_4_ (CFTS) synthesized for 1 week at 195 °C: (**A**) 1:0; (**B**) 7:1; (**C**) 3:1; (**D**) 5:3; (**E**) 1:1; (**F**) 3:5; (**G**) 1:3 EG:H_2_O ratio. With the increase in the water content in the initial batch, two generations of crystallites appear. The optimal EG:H_2_O ratio is 7:1.

The dependence of CFTS particle morphology on the EG:H_2_O ratio is apparent in SEM images (Figure 3). The products synthesized from EG:H_2_O ratios of 1:0 and 7:1 show spherical shapes (Figure 3A,B), whereas those synthesized from 3:1, 5:3, and 1:1 ratio show more irregular morphology (Figure 3C–E). The size of the CMTS particle is similar to that of CFTS (range = 2–3 μm). As the water content increases in the initial batch, two generations of crystallites appear that are characterized by more irregular edges and flower-like particles. This is shown in Figure 3G and F (EG:H_2_O ratio of 3:5 and 1:3). Larger, flower-like particles, relatively uniform in size (2–3 μm), are accompanied by much smaller spherical particles (about 0.1 μm).

### 3.2. Optimization of Time of Synthesis

Different times for the synthesis were studied to optimize the synthesis: 1 day, 3 days, 1 week, 2 weeks, and the synthesis repeated twice for 1 week each time in fresh solution. The temperature of the synthesis was maintained at 195 °C (except for 2 weeks experiment when the temperature was 160 °C for technical reasons) and the EG:H_2_O ratio was always equal to 7:1. 

**Figure 4:**
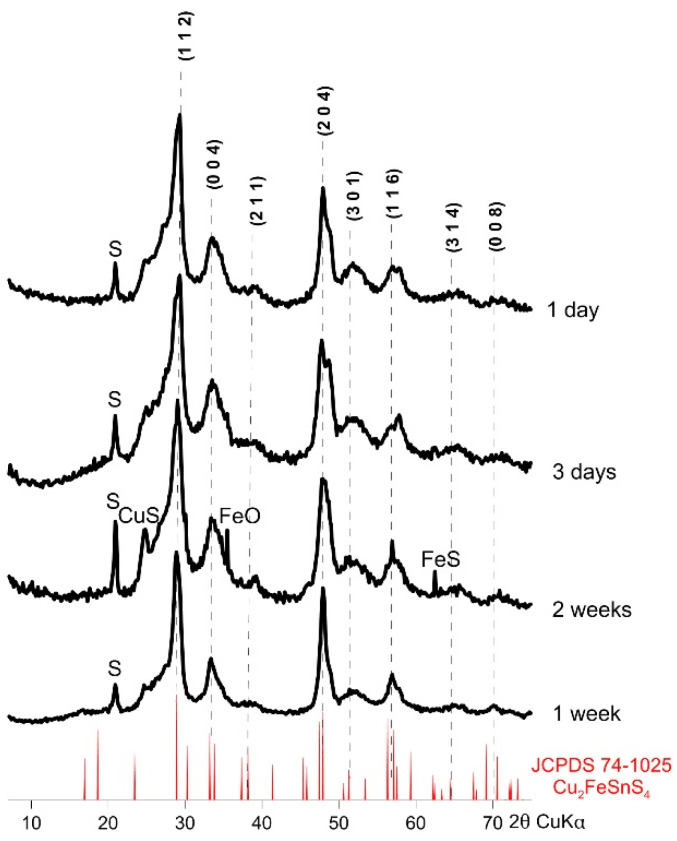
Powder X-ray diffraction patterns of synthetic Cu_2_FeSnS_4_ (CFTS) resulting from different synthesis times (synthesis at 195 °C and EG:H_2_O = 7:1). All experiments resulted in a tetragonal stannite identical to JCPDS standard No 74-1025. The optimal reaction time is one week.

Figure 4 shows the XRD patterns of the resulting CFTS particles. All experiments resulted in a tetragonal stannite identical to JCPDS standard No 74-1025. These patterns are similar in intensity and width of peaks. Small amounts of impurities (CuS, FeO, and FeS) were observed in samples synthesized for 2 weeks. Lower temperatures during synthesis (160 °C) also affected the product formation by decreasing crystallinity and forming impurities. This shows that 195 °C was an optimal temperature, although we found the formation of sulfur impurities in all the samples. After just one day of synthesis in 195 °C it is possible to obtain satisfactory purity and crystallinity of CFTS, but the optimal synthesis time is one week. There were no significant effects resulting from the prolongation of the time of synthesis of CFTS on XRD pattern. 

**Figure 5:**
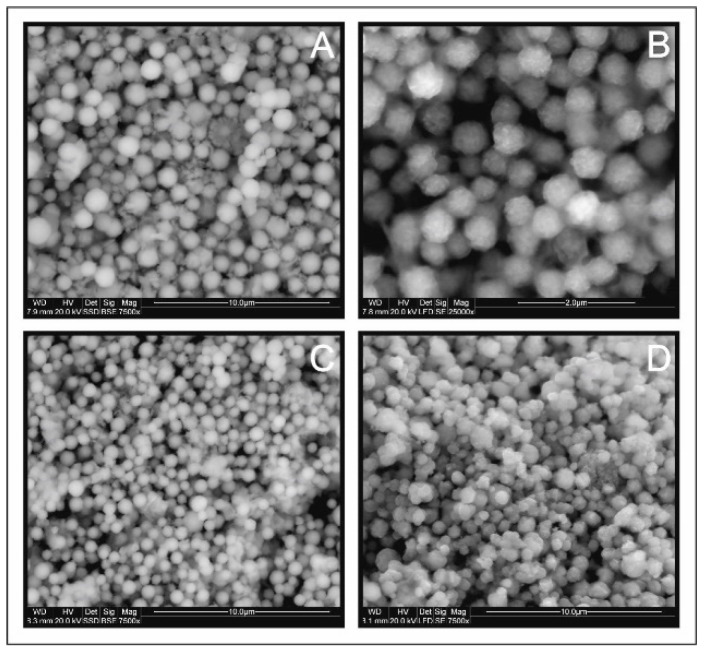
Scanning electron microscopy images of Cu_2_FeSnS_4_ (CFTS) resulting from different synthesis times: (**A**) 1 day, (**B**) 3 days, (**C**) 1 week, (**D**) 2 weeks. All products consisted of spherical particles of relatively uniform average size.

**Figure 6:**
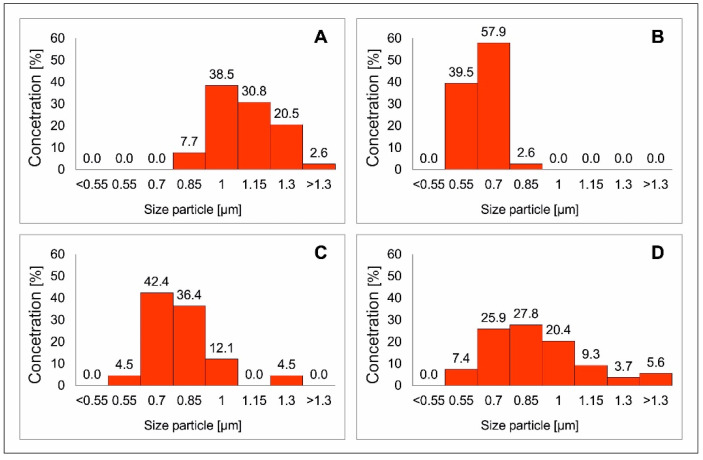
Statistical particle size distribution diagrams Cu_2_FeSnS_4_ (CFTS) resulting from different synthesis times: (**A**) 1 day, (**B**) 3 days, (**C**) 1 week, (**D**) 2 weeks. The extended synthesis time generates larger microparticles and the optimal reaction time is one week.

Figure 5 shows the SEM images of CFTS microspheres synthesized at different times. All products consisted of spherical particles of relatively average size from 0.6 to 1.5 µm (Figure 6). No changes were observed in the morphology of CFTS microparticles with extended synthesis time. This shows that CFTS microspheres of uniform particle size can be easily synthesized within 1 day. The exception is an experiment conducted twice for a week, which resulted in the two generations of sharp-edged particles.

**Figure 7:**
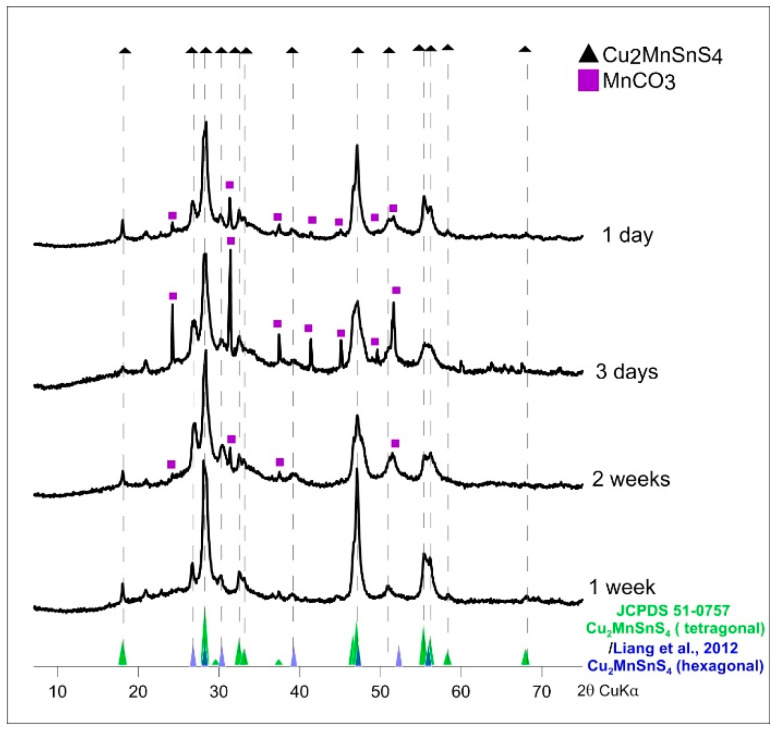
Powder X-ray diffraction patterns of synthetic Cu_2_MnSnS_4_ (CMTS) resulting from different synthesis times (synthesis at 195 °C and EG:H_2_O = 7:1). MnCO_3_ (rhodochrosite) was detected in the products of syntheses lasting one day and three days and in the product of synthesis at low temperature (160 °C for two weeks).

Figure 7 shows the XRD patterns for CMTS synthesized for different times. The dominant peaks are those corresponding to the tetragonal structure (JCPDS sheet No. 51-0757) can be observed at 2θ values ~18.02, 28.21, 29.56, 32.47, 33.07, 37.37, 44.46, 46.99, and 55.31. However, peaks corresponding to the hexagonal structure [42] can also be observed at 2θ values ~26.77, 28.21, 30.32, 39.23, 47.23, 52.26 and 55.89. In addition, small amounts of MnCO_3_ (rhodochrosite) were detected as undesirable contamination in the product of syntheses lasting 1 day and 3 days and in the product of synthesis at low temperature (160 °C for 2 weeks). 

**Figure 8:**
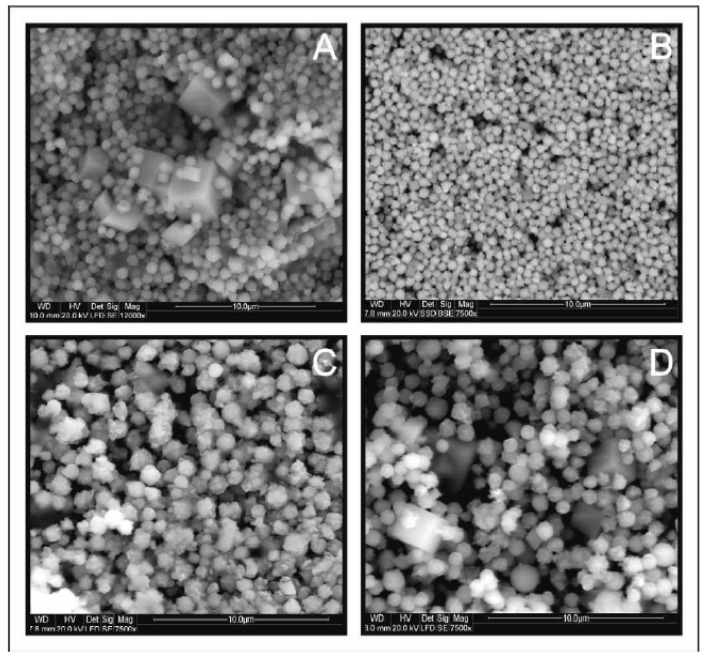
Scanning electron microscopy images for Cu_2_MnSnS_4_ (CMTS) resulting from different times of synthesis: (**A**) 1 day, (**B**) 3 days, (**C**) 1 week, (**D**) 2 weeks. The particle size depends on the synthesis time and the optimal reaction time is one week.

Figure 8 shows SEM images of CMTS depending on the duration of the synthesis. As a result of the shorter time of synthesis (1 day and 3 days), small microparticles of uniform spherical shape with an average size of 0.5–1 µm are produced. Larger microspheres of 1 to 1.5 µm diameter are formed during a long time of synthesis (1 and 2 weeks). 

**Figure 9:**
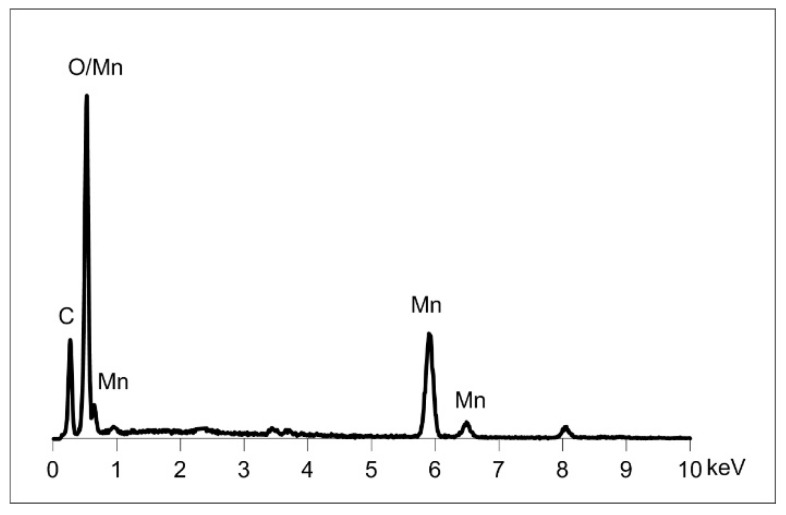
SEM/EDS spectrum showing the elemental composition of sparse admixtures of rhodochrosite (MnCO_3_) found in CMTS the products of syntheses lasting 1 day, 3 days, and 2 times a week.

In the products of the syntheses lasting 1 day, 3 days, and twice a week, the presence of an additional phase in the form of larger isometric crystals was observed. The result of elementary EDS (Figure 9) analysis is consistent with the composition of rhodochrosite, which is in line with the results of XRD analysis. 

### 3.3. Synthesis of Cu_2_(Mn_1−x_Fe_x_)SnS_4_ Solid Solution Series

**Figure 10:**
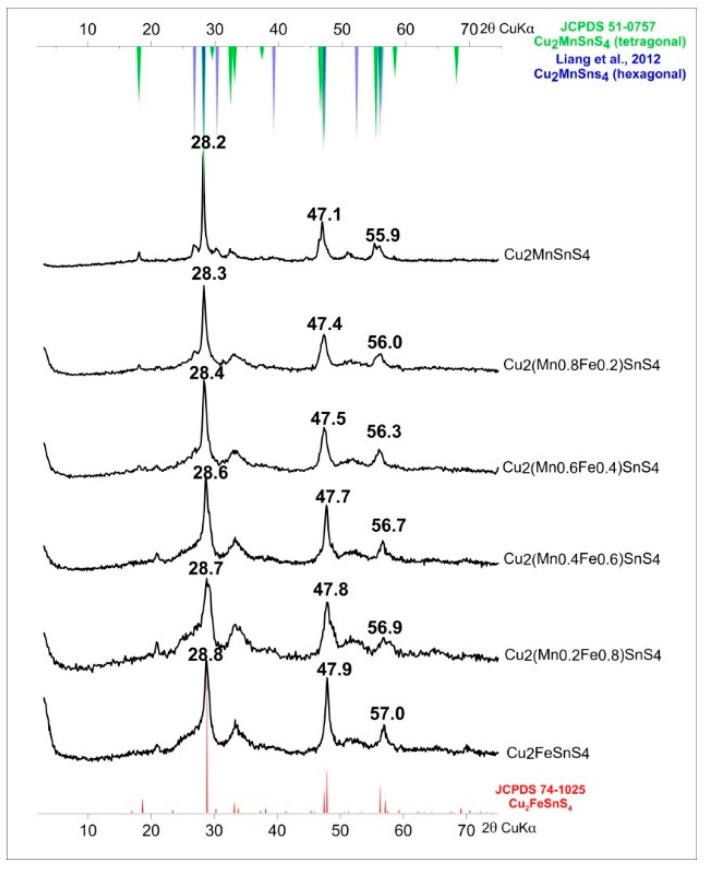
Powder X-ray diffraction patterns of Cu_2_(Mn_1−x_Fe_x_)SnS_4_ solid solution series. The systematic shift in the position of diffraction peaks towards lower angles 2θ with increasing Mn content is observed.

**Figure 11:**
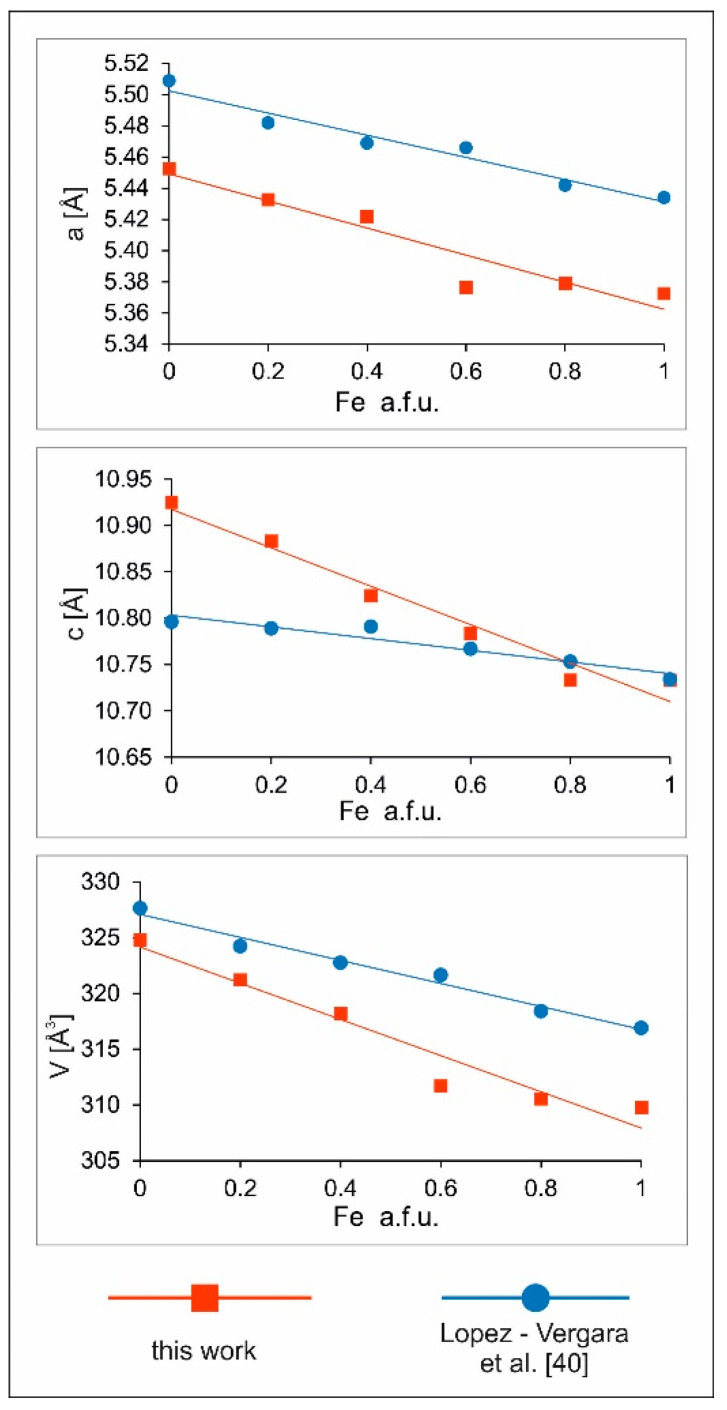
Linear changes of unit cell parameters in the CMTS-CFTS series as compared to similar results obtained by Lopez–Vergara et al. [40].

For the synthesis of solid solutions of Cu_2_(Mn_1−x_Fe_x_)SnS_4_ series, the synthesis time and EG:H_2_O ratio have been chosen to obtain the best crystalline products with the least amount of impurities. The EG:H_2_O ratio of 7:1 and a reaction time of one week at 195 °C met these expectations. The diffraction peaks for both this product and CFTS were the sharpest and most intense. The results are shown in Figure 10. Similar to Lopez-Vergara et al. [40], all samples showed tetragonal structure although the synthesis of CMTS under these conditions resulted in a product composed of a mixture of two phases (tetragonal and hexagonal). The unit cell parameters increased linearly with the increase in Mn content (Figure 11). This is manifested by the systematic shift in the position of diffraction peaks toward lower angles 2θ (from 28.8° 2θ to 28.2° 2θ for the main peak). In addition, the peaks became sharper, indicating an improvement in crystallinity. As the Mn content decreased, the hexagonal phase in the CMTS mixture disappeared. The peaks associated with the hexagonal structure can be observed in samples with Mn:Fe ratios of 1:1, 0.8:0.2, and 0.6:0.4. This suggests that CMTS formed by this method of synthesis can easily form tetragonal and hexagonal forms, whereas CFTS is more likely to precipitate in tetragonal structure.

**Figure 12:**
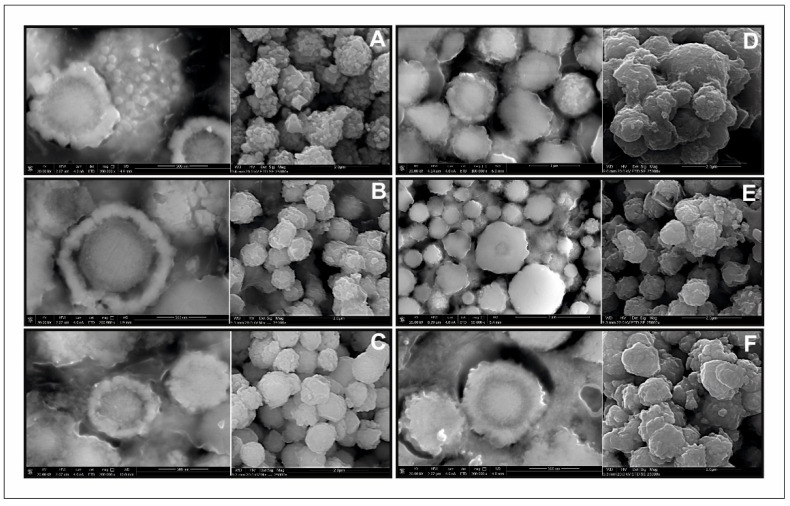
Scanning electron microscopy images of Cu_2_(Mn_1−x_Fe_x_)SnS_4_ solid solution series: (**A**) Cu_2_FeSnS_4_ (CFTS); (**B**) Mn_0.2_Fe_0.8_; (**C**) Mn_0.4_Fe_0.6_; (**D**) Mn_0.6_Fe_0.4_; (**E**) Mn_0.8_Fe_0.2_; (**F**) Cu_2_MnSnS_4_ (CMTS). The left image in each pair is taken from polished samples immersed in epoxy resin to show the inner nanostructure of the microspheres on the cross-sections, the right one is from raw powders.

**Figure 13:**
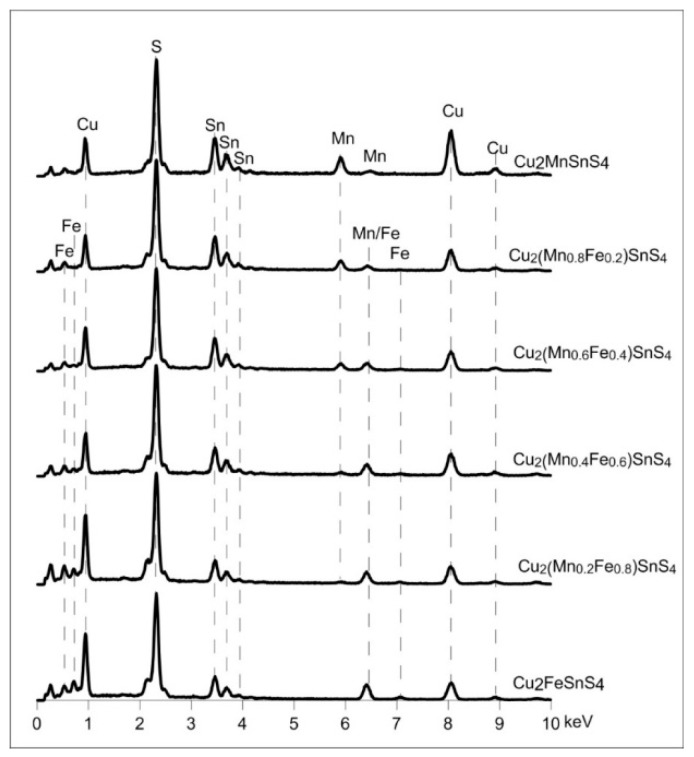
Elemental composition of Cu_2_(Mn_1−x_Fe_x_)SnS_4_ solid solution series. All the samples have the composition close to what was expected from the synthesis. All the samples have the composition close to what was expected from the synthesis.

The morphology of all samples in the series was very similar (Figure 12). They consisted of relatively uniform microspheres, ca. 1–1.5 μm in diameter, with a rough surface. The internal structure of the microspheres appeared to be concentric. The outer rim was made of aggregates of small crystals, whereas the core was much more homogeneous. The elemental composition of the members of the series as determined with the use of SEM/EDS microanalysis (Figure 13, Table 1) is very close to what was expected from the synthesis. This indicates that the composition of the synthesis can be precisely controlled by the conditions of the experiment. There was no difference in elemental composition between the rim and the core. However, with Mn-rich samples, it is hypothesized that the structure of the core and the rim may be different. Tiong et al. [39] reported that the material consisting of both hexagonal and tetragonal crystalline structures was obtained by using an organic sulfur precursor such as thioacetamide and Tu in the precursor solution of the hydrothermal reaction. They obtained pure tetragonal kesterite nanocrystals when Na_2_S was employed as the precursor of sulfur. A mixture of both tetragonal and hexagonal crystalline structure was obtained by using organic Tu as the precursor for sulfur [39,43,44].

**Figure 14:**
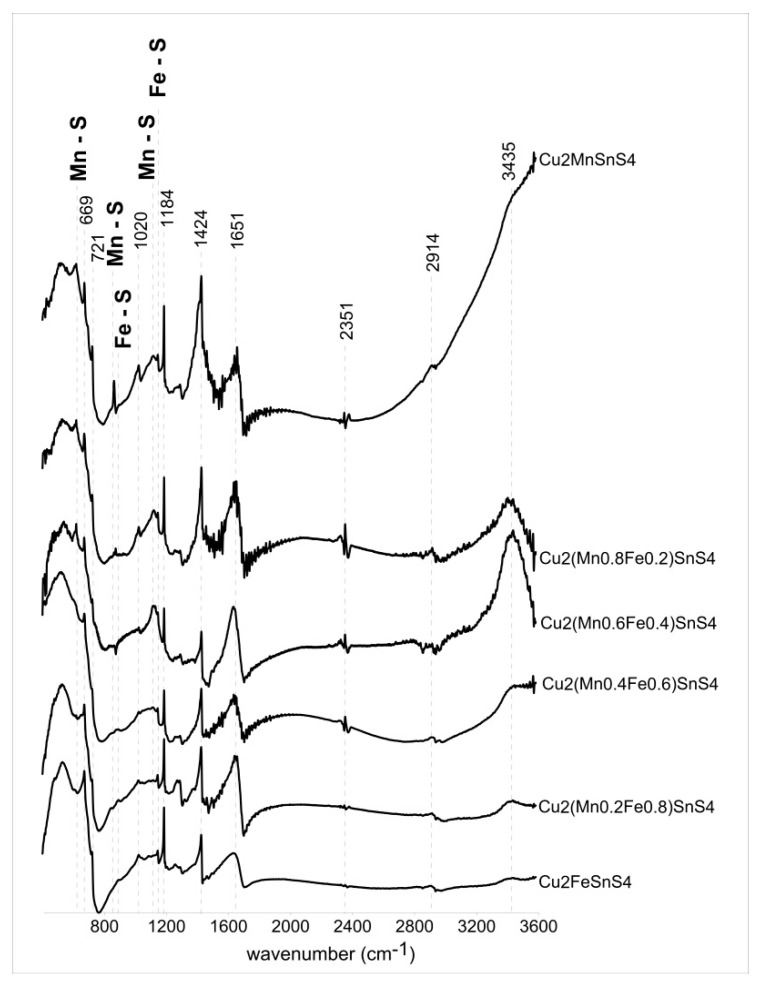
Fourier transform infrared spectra of Cu_2_(Mn_1−x_Fe_x_)SnS_4_ solid solution series. Several bands do not come directly from the synthesis products, but are an artifact of a small amount of impurity coming from the unreacted components used in the experiments. Specific Mn–S vibrations (617, 860, 1114 cm^−1^) and Fe–S vibrations (880 and 1142 cm^−1^) are marked.

Figure 14 shows the infrared absorption spectra for the six members of the CFTS–CMTS series from 400 to 3600 cm^−1^. Table 2 shows the band assignments and literature sources. Samples of Cu_2_MnSnS_4_, Cu_2_(Mn_0.8_Fe_0.2_)SnS_4_, and Cu_2_(Mn_0.6_Fe_0.4_)SnS_4_ show characteristic peaks at 617, 860, and 1114 cm^−1^ associated with specific Mn–S vibrations. These bands are assigned to the resonance interaction between the vibration modes of the sulfide ions in the crystal. As the Fe content increases, these bands disappear and bands at 880 and 1142 cm^−1^ show up which corresponds to Fe–S specific vibrations (samples Cu_2_(Mn_0.4_Fe_0.6_)SnS_4_, Cu_2_(Mn_0.2_Fe_0.8_)SnS_4_, and Cu_2_FeSnS_4_). The intensity of the bands is not very high, but their position is clear. FTIR analysis is very sensitive and allows for the identification of small amounts of contaminants. Therefore, in the presented spectra, many bands do not come directly from the synthesis products, but are an artifact of a small amount of impurity arising from the unreacted components used in the experiments. These reagents are not crystalline and were not detected in XRD analysis. The absorption peak at 3435 cm^–1^ corresponds to the presence of water, probably from the presence of humidity in the air. Similarly, small amount of CO_2_ adsorbed from the air results in bands at 2351 cm^–1^. The characteristic bands at 720 cm^–1^ are assigned to metal–O–H bending vibrations. The vibration band observed at 2935 cm^–1^ refers to the tensile strength of C–H bond, whereas the band at 1020 cm^–1^ corresponds to the tensile strength of C–S bond. The bands observed at 1651, 1424, and 1183 cm^–1^ were assigned to metal-Tu complexes. A more complete assignment of these peaks was proposed based on the study performed on metal–Tu complexes involving δN–H bending deformation (1651 cm^−1^), NH_2_ rocking vibration, C–S stretching vibration or N–C–N stretching vibration (1424 cm^−1^), C=S, C–N, or NH_2_ rocking vibration (1185 cm^−1^). The C=S stretching vibration mode of the Tu molecule is found at 668 cm^−1^.

**Figure 15:**
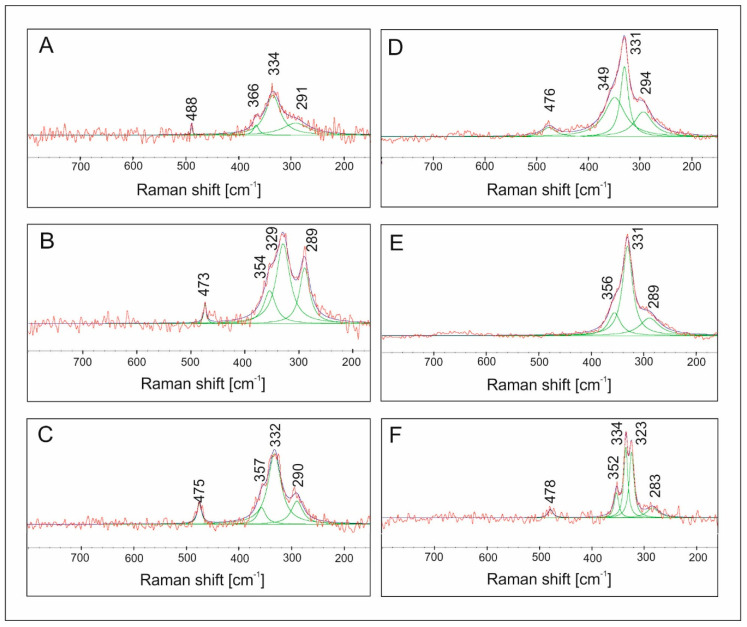
Raman spectra of: (**A**) Cu_2_FeSnS_4_, (**B**) Cu_2_(Mn_0.2_Fe_0.8_)SnS_4_, (**C**) Cu_2_(Mn_0.4_Fe_0.6_)SnS_4_, (**D**) Cu_2_(Mn_0.6_Fe_0.4_)SnS_4_, (**E**) Cu_2_(Mn_0.8_Fe_0.2_)SnS_4_, (**F**) Cu_2_MnSnS_4_. There is a variation in the frequency of the characteristic band of stannite: a main vibration mode at ~330 cm^−1^.

The Raman spectra of the samples analyzed (Figure 15) show bands characteristic of stannite in the range of 200–500 cm^−1^ [54]. Experimental Raman scattering spectra were analyzed by the deconvolution to Lorentzian curves. The dominant structure at about ~330 cm^−1^ in the experimental spectra is the vibration of pure anion mode of S anions around solid Sn cations, whereas the mode at about ~290 cm^−1^ can be considered as the pure S anion mode around Cu cation. Deconvolution shows an additional weaker band at 352–366 cm^−1^_,_ which is in good agreement with the frequencies reported for this compound [54]. As the Mn content in the samples increases, shifts in positions of some bands toward lower wavenumbers are observed (from 366 to 352 and from 291 to 283 cm^−1^). This may be related to a slight change in the length and force of the length of the metal–S bond or due to the two kinds of anionic mode of S anions around fixed Sn cations related to a different structure in a single crystal [55]. There is also a variation in the frequency of the main A1 vibration mode at ~330 cm^−1^. The broadening of the main peaks found in the solid solution with the increasing content of Fe is attributed to disorder effects due to chemical substitutions in M crystallographic positions [56]. The spectrum of CMTS (Figure 12F) shows the splitting of the main peak into 323 and 334 cm^−1^. This is consistent with XRD results. Typically, the main Raman peak of stannite structure CZTS is reported at 330–339 cm^−1^ matching the values well reported for crystals and films. Nevertheless, frequencies in the range of 320–334 cm^−1^ can also be found in the literature for CZTS in the same range where the strongest Raman features of wurtzite and stannite CZTS were often reported [57]. 

## 4. Conclusions

In this study, a low-cost and environmentally friendly method of preparation of semiconductors was successfully tested. The hydrothermal synthesis method, requiring simple reactants, and relatively low temperature and reasonable time can be used to obtain CFTS, CMTS, and their solid solutions on a larger scale. Optimal conditions for the synthesis are as follows: A mixture of H_2_O:EG at the ratio of 1:7 as the reaction medium, PVP as the surface ligand, and temperature of 195 °C for 7 days. The synthesis product precipitates as aggregates of nanocrystals. The aggregates form uniform, often concentric microspheres that are 1–1.5 μm in diameter. The chemical composition of the product can be well controlled by the chemical composition of the reactants, and the compound Cu_2_(Mn_1−x_Fe_x_)SnS_4_ forms a continuous solid solution series. Lopez-Vergara et al. [40] have drawn similar conclusions. If the series members rich in Fe are produced, then the product has a tetragonal structure. However, if the series members rich in Mn are produced, then the product is a mixture of phases with a tetragonal and hexagonal structure. We believe this material is a good candidate for the alternative at semiconductor and can be used as an adequate absorber in solar cells or catalyzation in photocatalysis.

## Figures and Tables

**Table 1:** Chemical composition (wt%) of Cu_2_(Mn_1−x_Fe_x_)SnS_4_ microcrystals from SEM/EDS analysis.

Cu	Mn	Fe	Sn	S	Estimated	Nominal
26.51	26.44	0.00	8.07	39.01	Cu_3.5_Mn_0.8_Sn_1.1_S_4_	Cu_2_MnSnS_4_
24.78	31.37	6.79	3.22	33.84	Cu_2.8_(Mn_0.6_Fe_0.3_)Sn_1.4_S_4_	Cu_2_(Mn_0.8_Fe_0.2_)SnS_4_
27.85	29.40	4.97	6.01	31.77	Cu_2.3_(Mn_0.4_Fe_0.5_)Sn_1.1_S_4_	Cu_2_(Mn_0.6_Fe_0.4_)SnS_4_
30.83	22.51	1.54	9.11	36.01	Cu_2.4_(Mn_0.1_Fe_0.7_)Sn_0.8_S_4_	Cu_2_(Mn_0.4_Fe_0.6_)SnS_4_
28.94	25.69	1.79	11.24	32.35	Cu_2.3_(Mn_0.1_Fe_0.9_)Sn_1_S_4_	Cu_2_(Mn_0.2_Fe_0.8_)SnS_4_
29.45	0.00	26.42	11.93	32.19	Cu_2.2_Fe_0.9_Sn_1_S_4_	Cu_2_FeSnS_4_

**Table 2:** Fourier transform infrared frequency range and functional groups of Cu_2_(Mn_1−x_Fe_x_)SnS_4_ solid solution series.

Cu_2_MnSnS_4_	Cu_2_(Mn_0.8_Fe_0.2_)SnS_4_	Cu_2_(Mn_0.6_Fe_0.4_)SnS_4_	Cu_2_(Mn_0.4_Fe_0.6_)SnS_4_	Cu_2_(Mn_0.2_Fe_0.8_)SnS_4_	Cu_2_FeSnS_4_	Interpretation	Literature
617	617	617	x	x	x	Mn-S specific vibrations	[45,46,47,48]
669	669	669	669	669	669	metal–thiourea complex – C–S stretching vibration	[49]
721	720	721	719	720	719	metal–O–H vibration	[50]
861	872	860	x	x	x	attributed to the resonance interaction between vibrational modes of sulfide ions in the crystal	[46,47]
x	x	x	880	885	890	attributed to the resonance interaction between vibrational modes of sulfide ions in the crystal - Fe–S specific vibrations	[46,47,50]
1020	1020	1021	1020	1020	1020	C-S stretching vibration	[49]
1114	1119	1117	x	x	x	Mn-S specific vibrations	[48]
x	x	x	1142	1142	1142	Fe-S specific vibrations	[50]
1184	1184	1184	1184	1184	1184	metal–thiourea complex - NH_2_ rocking vibration	[48,51]
1424	1424	1424	1424	1423	1424	metal–thiourea complex – N-C–N stretching and NH_2_ bending vibrational mode	[49,52]
1651	1649	1647	1636	1636	1632	metal–thiourea complex – N–C–N stretching and NH_2_ bending vibrational mode	[49,52]
2351	2351	2348	2351	2351	2351	CO_2_—was not related to the samples	[45]
2914	2913	2905	2909	2897	2897	C–H asymmetric stretching vibration	[45]
3435	3435	3435	3435	3435	3435	attributed to the O–H stretching vibration of H_2_O	[47,51,53]
